# 2-[(1,3-Benzothia­zol-2-yl)imino­meth­yl]-4-bromo­phenol

**DOI:** 10.1107/S160053681101275X

**Published:** 2011-04-13

**Authors:** Hai-Peng Diao, Ti-Jian Sun, Wen Liu

**Affiliations:** a Department of Chemistry, Shanxi Medical University, Taiyuan, Shanxi 030001, People’s Republic of China

## Abstract

In the title compound, C_14_H_9_BrN_2_OS, the dihedral angle between the benzene rings is 3.1 (3)°. An intra­molecular O—H⋯N(imine) hydrogen bond occurs. The crystal structure is stabilized by weak inter­molecular C—H⋯O inter­actions.

## Related literature

For the uses of Schiff bases, see: Da Silva *et al.* (2011 ▶); Dhar & Taploo (1982 ▶); Przybylski *et al.* (2009 ▶); Guo *et al.* (2007 ▶); Bringmann *et al.* (2004 ▶). For the structures of closely related imines, see: Liu *et al.* (2009 ▶); Asiri *et al.* (2010 ▶).
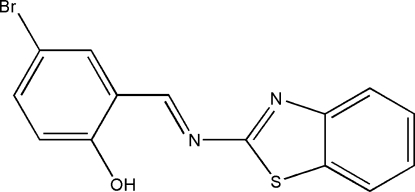


## Experimental

### 

#### Crystal data

C_14_H_9_BrN_2_OS
*M* *_r_* = 333.20
Monoclinic, 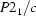

*a* = 26.1607 (2) Å
*b* = 4.0565 (2) Å
*c* = 12.1435 (3) Å
β = 91.5720 (1)°
*V* = 1288.19 (7) Å^3^
*Z* = 4
Mo *K*α radiation
μ = 3.34 mm^−1^
*T* = 296 K
0.45 × 0.40 × 0.38 mm

#### Data collection

Bruker SMART APEX CCD area-detector diffractometer
Absorption correction: multi-scan (*SADABS*; Bruker, 2001 ▶) *T*_min_ = 0.242, *T*_max_ = 0.281
11848 measured reflections
2232 independent reflections
1466 reflections with *I* > 2σ(*I*)
*R*_int_ = 0.138

#### Refinement

*R*[*F* ^2^ > 2σ(*F* ^2^)] = 0.045
*wR*(*F* ^2^) = 0.118
*S* = 0.93
2232 reflections
173 parameters
H-atom parameters constrained
Δρ_max_ = 0.36 e Å^−3^
Δρ_min_ = −0.54 e Å^−3^

### 

Data collection: *SMART* (Bruker, 2001 ▶); cell refinement: *SAINT* (Bruker, 2001 ▶); data reduction: *SAINT*; program(s) used to solve structure: *SHELXTL* (Sheldrick, 2008 ▶); program(s) used to refine structure: *SHELXTL*; molecular graphics: *SHELXTL*; software used to prepare material for publication: *SHELXTL*.

## Supplementary Material

Crystal structure: contains datablocks I, global. DOI: 10.1107/S160053681101275X/bh2345sup1.cif

Structure factors: contains datablocks I. DOI: 10.1107/S160053681101275X/bh2345Isup2.hkl

Additional supplementary materials:  crystallographic information; 3D view; checkCIF report

## Figures and Tables

**Table 1:** Hydrogen-bond geometry (Å, °)

*D*—H⋯*A*	*D*—H	H⋯*A*	*D*⋯*A*	*D*—H⋯*A*
O1—H1⋯N1	0.82	1.87	2.600 (6)	148
C7—H7⋯O1^i^	0.93	2.47	3.345 (6)	157

Symmetry code: (i) 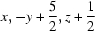.

**Fig. 1.**
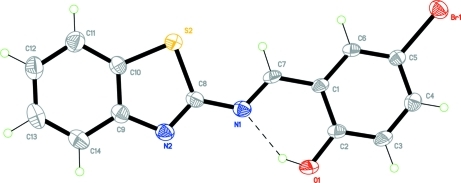
A view of the structure of the title compound with displacement ellipsoids drawn at the 30% probability level.

**Fig. 2.**
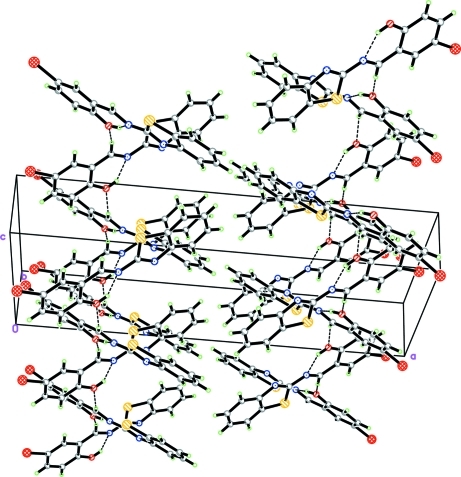
Crystal packing of the title compound. Hydrogen bonds are shown as dashed lines.

## supplementary crystallographic information

### Comment

Schiff bases are some of the most widely used organic compounds. They are used as pigments and dyes, catalysts, intermediates in organic synthesis, and as polymer stabilisers (Da Silva *et al.*, 2011). They have also been shown to exhibit a broad range of biological activities, including antifungal, antibacterial, antimalarial, antiproliferative, anti-inflammatory, antiviral, and antipyretic properties (Dhar & Taploo, 1982; Przybylski *et al.*, 2009). The imine group present in such compounds has been shown to be critical to their biological activities (Guo *et al.*, 2007; Bringmann *et al.*, 2004). It was thus of interest to synthesize the title compound.

The X-ray structural analysis confirmed the assignment of the structure of the title compound (Fig. 1). The bond length of C8—N1 is 1.396 (6) Å, which is shorter than normal C—N [1.47 Å]. The dihedral angle between the two benzene rings (C1···C6 and C9···C14) is 3.1 (3)°, it is a little larger than 2.81 (9)° or 2.6 (1)° found in a related structure (Liu *et al.*, 2009; Asiri *et al.*, 2010). In the crystal structure (Fig. 2), the compound is further stabilized by intramolecular O—H···N and weak intermolecular C—H···O hydrogen-bond interactions.

### Experimental

10 mL of 5 mmol 5-bromo-2-hydroxybenzaldehyde ethanol solution was added to 10 mL of the 5 mmol (0.7515 g) of 2-aminobenzothiazole ethanol solution. The resulting solution was refluxed for about 3 h, and then cooled to room temperature. Yellow crystals of title compound were obtained after 2 weeks of slow evaporation of the filtrate at room temperature.

### Refinement

H atoms attached to C and O atoms were placed in geometrically idealized positions with C*sp*^2^—H = 0.93 Å and O—H = 0.82 Å. The isotropic displacement parameters for H atoms were fixed as *U*_iso_(H) = 1.2*U*_eq_(carrier C atom) and *U*_iso_(H1) = 1.5*U*_eq_(O1).

### Crystal data

**Table:**

C_14_H_9_BrN_2_OS	*Z* = 4
*M**_r_* = 333.20	*F*(000) = 664
Monoclinic, *P*2_1_/*c*	*D*_x_ = 1.718 Mg m^−^^3^
Hall symbol: -P 2ybc	Mo *K*α radiation, λ = 0.71073 Å
*a* = 26.1607 (2) Å	θ = 3.4–21.4°
*b* = 4.0565 (2) Å	µ = 3.34 mm^−^^1^
*c* = 12.1435 (3) Å	*T* = 296 K
β = 91.5720 (1)°	Block, yellow
*V* = 1288.19 (7) Å^3^	0.45 × 0.40 × 0.38 mm

### Data collection

**Table:**

Bruker SMART APEX CCD area-detector diffractometer	2232 independent reflections
Radiation source: fine-focus sealed tube	1466 reflections with *I* > 2σ(*I*)
graphite	*R*_int_ = 0.138
φ and ω scans	θ_max_ = 25.0°, θ_min_ = 1.6°
Absorption correction: multi-scan (*SADABS*; Bruker, 2001)	*h* = −30→30
*T*_min_ = 0.242, *T*_max_ = 0.281	*k* = −4→4
11848 measured reflections	*l* = −14→14

### Refinement

**Table:**

Refinement on *F*^2^	Primary atom site location: structure-invariant direct methods
Least-squares matrix: full	Secondary atom site location: difference Fourier map
*R*[*F*^2^ > 2σ(*F*^2^)] = 0.045	Hydrogen site location: inferred from neighbouring sites
*wR*(*F*^2^) = 0.118	H-atom parameters constrained
*S* = 0.93	*w* = 1/[σ^2^(*F*_o_^2^)] where *P* = (*F*_o_^2^ + 2*F*_c_^2^)/3
2232 reflections	(Δ/σ)_max_ < 0.001
173 parameters	Δρ_max_ = 0.36 e Å^−^^3^
0 restraints	Δρ_min_ = −0.54 e Å^−^^3^
0 constraints	

### Fractional atomic coordinates and isotropic or equivalent isotropic displacement parameters (Å^2^)

**Table:**

	*x*	*y*	*z*	*U*_iso_*/*U*_eq_	
Br1	0.03658 (2)	1.69312 (15)	0.65038 (5)	0.0536 (2)	
S2	0.29866 (5)	0.7609 (3)	0.70906 (12)	0.0488 (4)	
C1	0.17288 (19)	1.3148 (12)	0.5361 (4)	0.0370 (11)	
O1	0.20795 (15)	1.3280 (10)	0.3543 (3)	0.0582 (10)	
H1	0.2300	1.2208	0.3877	0.087*	
C12	0.4377 (2)	0.3239 (15)	0.7216 (6)	0.0633 (17)	
H12	0.4606	0.2193	0.7700	0.076*	
C4	0.0898 (2)	1.6691 (13)	0.4506 (4)	0.0458 (13)	
H4	0.0620	1.7888	0.4225	0.055*	
C6	0.13249 (18)	1.4049 (11)	0.6017 (4)	0.0372 (12)	
H6	0.1335	1.3473	0.6758	0.045*	
N2	0.33356 (16)	0.7896 (11)	0.5088 (4)	0.0471 (11)	
N1	0.25338 (15)	1.0410 (11)	0.5215 (3)	0.0432 (10)	
C2	0.17096 (19)	1.4091 (12)	0.4229 (4)	0.0399 (12)	
C8	0.29517 (19)	0.8711 (13)	0.5676 (4)	0.0437 (13)	
C5	0.09127 (19)	1.5761 (12)	0.5601 (4)	0.0381 (12)	
C7	0.21554 (18)	1.1329 (12)	0.5801 (4)	0.0386 (12)	
H7	0.2160	1.0782	0.6545	0.046*	
C9	0.3704 (2)	0.6300 (13)	0.5745 (5)	0.0469 (14)	
C3	0.1294 (2)	1.5847 (13)	0.3824 (4)	0.0501 (14)	
H3	0.1279	1.6472	0.3087	0.060*	
C11	0.3918 (2)	0.4411 (14)	0.7602 (5)	0.0596 (16)	
H11	0.3837	0.4206	0.8340	0.071*	
C10	0.35821 (19)	0.5906 (12)	0.6847 (4)	0.0432 (13)	
C13	0.4499 (2)	0.3597 (14)	0.6131 (6)	0.0653 (18)	
H13	0.4812	0.2816	0.5897	0.078*	
C14	0.4168 (2)	0.5088 (15)	0.5376 (5)	0.0581 (16)	
H14	0.4253	0.5279	0.4640	0.070*	

### Atomic displacement parameters (Å^2^)

**Table:**

	*U*^11^	*U*^22^	*U*^33^	*U*^12^	*U*^13^	*U*^23^
Br1	0.0431 (3)	0.0658 (4)	0.0521 (4)	0.0039 (3)	0.0053 (2)	−0.0054 (3)
S2	0.0435 (8)	0.0614 (9)	0.0415 (8)	0.0046 (6)	0.0040 (6)	0.0028 (7)
C1	0.046 (3)	0.041 (3)	0.023 (3)	−0.001 (2)	−0.005 (2)	−0.003 (2)
O1	0.058 (2)	0.089 (3)	0.0284 (19)	0.013 (2)	0.0052 (18)	0.008 (2)
C12	0.046 (4)	0.064 (4)	0.080 (5)	0.001 (3)	−0.003 (3)	0.010 (4)
C4	0.043 (3)	0.050 (3)	0.044 (3)	−0.001 (3)	−0.010 (3)	−0.001 (3)
C6	0.042 (3)	0.045 (3)	0.025 (3)	−0.006 (2)	0.001 (2)	−0.004 (2)
N2	0.043 (3)	0.060 (3)	0.039 (3)	−0.004 (2)	0.002 (2)	−0.005 (2)
N1	0.040 (2)	0.054 (3)	0.036 (2)	−0.006 (2)	0.001 (2)	−0.004 (2)
C2	0.043 (3)	0.050 (3)	0.027 (3)	−0.007 (2)	0.001 (2)	−0.003 (2)
C8	0.039 (3)	0.054 (3)	0.038 (3)	−0.005 (3)	0.005 (2)	−0.004 (3)
C5	0.042 (3)	0.038 (3)	0.034 (3)	−0.003 (2)	−0.003 (2)	−0.002 (2)
C7	0.044 (3)	0.050 (3)	0.021 (3)	−0.008 (2)	−0.003 (2)	0.002 (2)
C9	0.039 (3)	0.050 (3)	0.052 (3)	−0.008 (3)	0.003 (3)	−0.004 (3)
C3	0.056 (4)	0.067 (4)	0.027 (3)	0.003 (3)	−0.007 (3)	0.005 (3)
C11	0.054 (4)	0.065 (4)	0.060 (4)	−0.001 (3)	−0.003 (3)	0.004 (3)
C10	0.036 (3)	0.043 (3)	0.051 (3)	−0.006 (2)	0.002 (3)	−0.002 (3)
C13	0.042 (3)	0.060 (4)	0.094 (5)	0.002 (3)	0.006 (4)	−0.012 (4)
C14	0.051 (4)	0.065 (4)	0.059 (4)	−0.008 (3)	0.012 (3)	−0.011 (3)

### Geometric parameters (Å, °)

**Table:**

Br1—C5	1.887 (5)	C6—H6	0.9300
S2—C10	1.737 (5)	N2—C8	1.291 (6)
S2—C8	1.775 (5)	N2—C9	1.393 (7)
C1—C6	1.390 (6)	N1—C7	1.290 (6)
C1—C2	1.426 (6)	N1—C8	1.396 (6)
C1—C7	1.430 (7)	C2—C3	1.380 (7)
O1—C2	1.335 (5)	C7—H7	0.9300
O1—H1	0.8200	C9—C10	1.393 (7)
C12—C13	1.372 (9)	C9—C14	1.395 (7)
C12—C11	1.384 (8)	C3—H3	0.9300
C12—H12	0.9300	C11—C10	1.392 (7)
C4—C5	1.382 (7)	C11—H11	0.9300
C4—C3	1.387 (7)	C13—C14	1.382 (8)
C4—H4	0.9300	C13—H13	0.9300
C6—C5	1.367 (6)	C14—H14	0.9300
			
C10—S2—C8	87.6 (2)	C6—C5—Br1	121.1 (4)
C6—C1—C2	118.3 (5)	C4—C5—Br1	119.2 (4)
C6—C1—C7	121.3 (4)	N1—C7—C1	123.1 (4)
C2—C1—C7	120.4 (4)	N1—C7—H7	118.5
C2—O1—H1	109.5	C1—C7—H7	118.5
C13—C12—C11	121.0 (6)	C10—C9—N2	115.5 (5)
C13—C12—H12	119.5	C10—C9—C14	119.5 (6)
C11—C12—H12	119.5	N2—C9—C14	125.0 (5)
C5—C4—C3	120.3 (5)	C2—C3—C4	120.6 (5)
C5—C4—H4	119.8	C2—C3—H3	119.7
C3—C4—H4	119.8	C4—C3—H3	119.7
C5—C6—C1	121.8 (5)	C12—C11—C10	117.7 (6)
C5—C6—H6	119.1	C12—C11—H11	121.2
C1—C6—H6	119.1	C10—C11—H11	121.2
C8—N2—C9	109.8 (4)	C11—C10—C9	121.7 (5)
C7—N1—C8	121.7 (4)	C11—C10—S2	127.9 (4)
O1—C2—C3	118.9 (4)	C9—C10—S2	110.4 (4)
O1—C2—C1	121.9 (5)	C12—C13—C14	121.7 (6)
C3—C2—C1	119.2 (5)	C12—C13—H13	119.1
N2—C8—N1	121.2 (5)	C14—C13—H13	119.1
N2—C8—S2	116.7 (4)	C13—C14—C9	118.4 (6)
N1—C8—S2	122.1 (4)	C13—C14—H14	120.8
C6—C5—C4	119.7 (5)	C9—C14—H14	120.8
			
C2—C1—C6—C5	0.8 (7)	C8—N2—C9—C10	0.7 (6)
C7—C1—C6—C5	−179.3 (4)	C8—N2—C9—C14	−180.0 (5)
C6—C1—C2—O1	−179.1 (5)	O1—C2—C3—C4	178.9 (5)
C7—C1—C2—O1	1.1 (7)	C1—C2—C3—C4	0.0 (7)
C6—C1—C2—C3	−0.1 (7)	C5—C4—C3—C2	−0.4 (8)
C7—C1—C2—C3	−180.0 (5)	C13—C12—C11—C10	1.2 (9)
C9—N2—C8—N1	178.7 (4)	C12—C11—C10—C9	−1.6 (8)
C9—N2—C8—S2	−0.6 (6)	C12—C11—C10—S2	−179.6 (4)
C7—N1—C8—N2	−177.8 (5)	N2—C9—C10—C11	−178.8 (5)
C7—N1—C8—S2	1.5 (7)	C14—C9—C10—C11	1.8 (8)
C10—S2—C8—N2	0.3 (4)	N2—C9—C10—S2	−0.4 (6)
C10—S2—C8—N1	−179.0 (4)	C14—C9—C10—S2	−179.8 (4)
C1—C6—C5—C4	−1.3 (7)	C8—S2—C10—C11	178.3 (5)
C1—C6—C5—Br1	179.5 (4)	C8—S2—C10—C9	0.1 (4)
C3—C4—C5—C6	1.1 (7)	C11—C12—C13—C14	−1.0 (9)
C3—C4—C5—Br1	−179.7 (4)	C12—C13—C14—C9	1.2 (9)
C8—N1—C7—C1	178.3 (4)	C10—C9—C14—C13	−1.5 (8)
C6—C1—C7—N1	179.0 (4)	N2—C9—C14—C13	179.1 (5)
C2—C1—C7—N1	−1.1 (7)		

### Hydrogen-bond geometry (Å, °)

**Table:**

*D*—H···*A*	*D*—H	H···*A*	*D*···*A*	*D*—H···*A*
O1—H1···N1	0.82	1.87	2.600 (6)	148
C7—H7···O1^i^	0.93	2.47	3.345 (6)	157

Symmetry codes: (i) *x*, −*y*+5/2, *z*+1/2.
